# Prolonged systemic inflammation persistently modifies synaptic plasticity in the hippocampus: modulation by the stress hormones

**DOI:** 10.3389/fnmol.2013.00046

**Published:** 2013-12-04

**Authors:** Nicola Maggio, Efrat Shavit-Stein, Amir Dori, Ilan Blatt, Joab Chapman

**Affiliations:** ^1^Department of Neurology, The Joseph Sagol Neuroscience Center, The Chaim Sheba Medical CenterTel HaShomer, Israel; ^2^Talpiot Medical Leadership Program, The Chaim Sheba Medical CenterTel HaShomer, Israel; ^3^Department of Neurology, Washington University School of MedicineSaint Louis, MO, USA; ^4^Department of Neurology, Sackler Faculty of Medicine, Tel Aviv UniversityTel Aviv, Israel

**Keywords:** inflammation, hippocampus, synaptic plasticity, LTP, corticosterone, mineralocorticosteroid receptors, glucocorticosteroid receptors, LPS

## Abstract

Transient systemic inflammation has been shown to cause altered behavior both in humans and in laboratory animals through activation of microglia and heightened level of cytokines detected in the brain and in the body. Furthermore, both activated microglia and the increased cytokines level have been associated with the sudden clinical deterioration in demented people or in aged patients upon systemic inflammation. Whilst it is increasingly becoming clear the role of transient systemic inflammation in promoting dementia in aged individuals, it is still a matter of debate whether prolonged systemic inflammation might persistently modify the brain. In this study, we examined the influence of a systemic long term inflammatory event on synaptic plasticity. We report that while a short exposure to LPS produces transient deficit in long term potentiation (LTP) expression, systemic prolonged inflammation impairs LTP in slices of animals previously primed by a Complete Freund’s adjuvant injection. Interestingly, steroids are able to modulate this effect: whereas glucocorticosteroid (GR) activation further reduces LTP, mineralocorticosteroid receptors (MR) activation promotes the full recovery of LTP. We believe that this research advances the current understandings on the role of the immune system in the onset and progression of cognitive deficits following long lasting systemic inflammation, and proposes possible insights on future strategies in order to prevent early dementia in these predisposed individuals.

## INTRODUCTION

In health and disease, the immune system and the brain constantly communicate with each other. Specifically, in quiescent periods, the immune system positively regulates learning, memory, neural plasticity, and neurogenesis (Dantzer et al., 2008; Bitzer-Quintero and Gonzalez-Burgos, 2012; Kohman and Rhodes, 2013). In particular, during learning or high frequency stimulation, known to induce Long Term Potentiation (LTP), a cellular correlate of learning and memory (Maggio and Segal, 2010; Segal et al., 2010), microglia secretes several mediators which are thought to support plasticity (Bitzer-Quintero and Gonzalez-Burgos, 2012). Upon stimulation by endogenous (e.g., injury, stroke, autoimmune processes) or exogenous challenges (e.g., pathogens or severe psychological stressors), the immune system modifies the delicate neuro-glial interactive balance and impairs neuronal plasticity and memory (Besedovsky and del Rey, 2011). To date, however, it is still under investigation whether the activation of the immune system upon infection may cause permanent changes in neuronal functions (Semmler et al., 2007). In a young and healthy brain, acute confusional state is generally the first neurological manifestation due to systemic inflammation (Young, 1995). This is usually a transient condition, accompanied by changes in the EEG patterns together with an increase in pro-inflammatory cytokines and cortisol levels in the blood stream of patients (Isik and Caliskan, 2008; van den Boogaard et al., 2010). In old individuals, acute cognitive impairment commonly occurs in association with peripheral infection (Wofford et al., 1996; Chiovenda et al., 2002). In addition, in old persons an increased blood level of systemic inflammation markers is associated with the development of cognitive deficits (Trollor et al., 2012) and greater brain atrophy than expected for age (Jefferson et al., 2007). Similarly, in healthy aged mice, i.p. administration of lipopolysaccharide (LPS, mimicking bacterial endotoxin) results both in depression-like behaviors, deficits in hippocampal-dependent learning and memory (Godbout et al., 2008) as well as in exaggerated inflammatory cytokine response (Chen et al., 2008). This is thought to be due to “primed” microglia which may release excessive concentrations of inflammatory cytokines in the CNS upon receiving a triggering stimulus (i.d. LPS or peripheral infection; Dilger and Johnson, 2008). Finally, in chronic neurodegenerative diseases, systemic inflammation has been hypothesized to be involved in the pathogenesis of dementia (Sundelof et al., 2009) and thought to accelerate neurodegeneration and promote delirium (Cunningham et al., 2005; Murray et al., 2011). Altogether, while it is accepted that systemic inflammation underlies acute brain dysfunctions and neurological exacerbations in predisposed subjects, not much is known about the long term consequences of a persistent systemic inflammation.

Among others, systemic inflammation is known to activate the stress response and release of cortisol (corticosterone in rodents; Wiersinga, 2011). Furthermore, steroids are considered a milestone for the treatment of patients undergoing severe, prolonged inflammation (Patel and Balk, 2012; Sherwin et al., 2012). We and others have shown that corticosterone may either facilitate or depress synaptic plasticity and LTP based on the specific activation of its receptors subtypes, the mineralocorticosteroid receptors (MR) or the glucocorticosteroid receptors (GR), respectively (Alfarez et al., 2006; Joels and Krugers, 2007; Maggio and Segal, 2010; Krugers et al., 2011; Maggio and Segal, 2012c; Timmermans et al., 2013). While the association between systemic inflammation and steroids is clear, the consequences on synaptic plasticity upon activation of different steroids receptors in animals with systemic inflammation are yet unknown.

In this study, we tested the effects of short and long term systemic LPS treatment on LTP and its modulation by corticosteroid receptors. We believe that our findings add novel insights on the relationship between inflammation and synaptic plasticity and may help designing future therapeutic strategies to prevent the long term consequences of systemic inflammation.

## MATERIALS AND METHODS

### MICE AND TREATMENTS

Animal handling was approved by the Institutional Animal Care and Use Committee, which adheres to the national law, and NIH rules. Two-months old Balb/c mice underwent two treatment protocols: in one protocol, mice were exposed to i.p. injections of LPS (1 mg/Kg) twice a week for a week (short treatment). In the other one, animals received LPS injections for a month (twice a week; long treatment). In order to address whether the activation of the immune system prior to the LPS administration would affect LTP in a particular manner, we exposed some of the animals to a single Complete Freund’s Adjuvant (Adj) injection (diluted 1:1 in saline, 100 μL total volume injected/mouse) 24 h prior to the beginning of the LPS treatment. In total, we had four groups of animals (*n* = 8 animals/group at each time point, two slices/animal for each pharmacological treatment) undergoing either a short or a long treatment: one group was treated with LPS, and additional one received Adj, another group was injected with Adj + LPS, and finally the control untreated animals. The effects of stress hormones were studied by applying either Corticosterone (Cort, 100 nM), RU (500 nM) + Cort (MR activation) or Spironolactone (500nM) + Cort (GR activation) directly on the hippocampal slices, as previously described (Maggio and Segal, 2010). Application of MR or GR antagonists alone did not result in any effect compared to control untreated slices of the respective treatment group.

### ELECTROPHYSIOLOGY IN BRAIN SLICES

Extracellular recordings in hippocampal slices were performed as previously reported (Maggio and Segal, 2007b, 2011). Briefly, following anesthesia with ketamine/xylazine (dosage of 100 and 10 mg/Kg, respectively), animals were rapidly decapitated, the brain removed, and 400 μm slices prepared using a vibroslicer. Slices were incubated for 1.5 h in a humidified, carbogenated (5% CO_2_ and 95% O_2_) gas atmosphere at 33 ± 1°C and perfused with ACSF [containing (in micromolar) 124 NaCl, 2 KCl, 26 NaHCO_3_, 1.24 KH_2_PO_4_, 2.5 CaCl_2_, 2 MgSO_4_, and 10 glucose, pH 7.4] in a standard interface chamber. Recordings were made with a glass pipette containing 0.75 M NaCl (4 MΩ) placed in the stratum radiatum CA1. Stimulation was evoked using a pulse stimulator and delivered through a bipolar nichrome electrode. LTP was induced by high-frequency stimulation (HFS) consisting of 100 pulses at twice the test intensity, delivered at a frequency of 100 Hz (100 Hz, 1 s). Before applying the tetanic stimulation, baseline values were recorded at a frequency of 0.033 Hz. Responses were digitized at 5 kHz and stored on a computer. Off-line analysis and data acquisition was performed by Spike 2 software. All numerical data are expressed as mean ± SEM, and EPSP slope changes after tetanic stimulation were calculated with respect to baseline. There were no systematic differences in the magnitudes of the baseline responses in the different conditions. Unless otherwise indicated, statistical evaluations were performed by applying either one way or two ways ANOVA as the case may be, followed by a *post hoc* Tukey’s test, *p* values of 0.05 were considered a significant difference between means.

## RESULTS

### PROLONGED SYSTEMIC INFLAMMATION PERSISTENTLY MODIFIES SYNAPTIC PLASTICITY IN THE HIPPOCAMPUS

**FIGURE 1:**
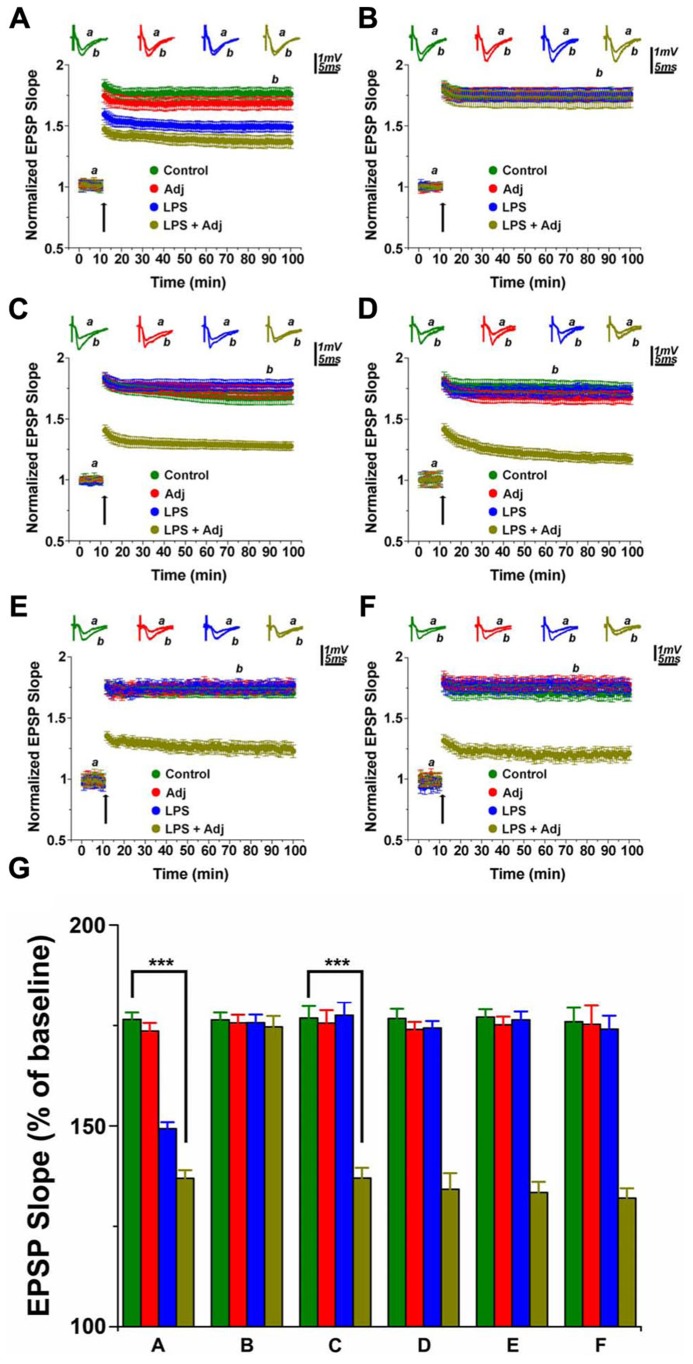
**LTP in hippocampal slices of animals undergoing LPS treatments of different duration.** LTP is impaired in animals treated with LPS and LPS + Adj for a week **(A)**. Upon interruption of the treatment for a week LTP is restored in LPS and LPS + Adj animals **(B)**. LTP is persistently impaired in animals treated with LPS + Adj for a month **(C)** even after interruption of treatment **(D)** 1 week upon treatment interruption, **(E)** 1 month following treatment interruption, **(F)** 2 months following treatment interruption. **(G)** A combined analysis of all the data presented, conducted at 85 min after the tetanic stimulation. Further details of the results and the statistical comparisons are described in the Section “Results.” Statistical analyses were made with two way analysis of variance, followed by *post hoc* Tukey’s comparisons. In each panel of data, the arrow indicates the time of HFS delivery, the top traces are sample illustrations of original records before (a) and after (b) tetanic stimulation. ***p < 0.001.

It is unclear whether exposure to a long lasting systemic inflammation may produce enduring changes in the brain. Here, we investigated the effects that two treatments with LPS (1 mg/Kg) of different duration might produce on LTP at the CA3-CA1 hippocampal synapses. In one treatment protocol, mice were exposed to i.p. injections of LPS twice a week for a week (short treatment), in the other one, animals received LPS injections for a month (twice a week; long treatment). To address whether the activation of the immune system prior to the LPS administration would have affected LTP in any manner, we exposed some of the animals to a single Adj injection 24 h prior to the beginning of the LPS treatment. In total, we had four groups of animals enrolled in the study: one that was treated with LPS, one that received Adj, another group received Adj + LPS, and finally the control untreated animals. A short treatment with LPS reduced LTP both in LPS and in Adj + LPS mice (1.49 ± 0.04 and 1.36 ± 0.04, respectively; values at 85 min of recordings) compared to Adj and control groups (1.67 ± 0.05 and 1.77 ± 0.04, respectively; values at 85 min of recordings; *F* = 10.71, *p* < 0.001, **Figure 1A**). Interestingly, LTP fully recovered in LPS and Adj + LPS animals 1 week upon interruption of LPS injections. Indeed, LTP in these animals reached similar values to those of the other groups. These data show that a short duration treatment with LPS affects LTP transiently: LTP is restored in a time frame of a week after the interruption of the LPS administration. However, a different phenomenon occurs if the treatment is being prolonged for a month. Indeed, LTP was impaired in Adj + LPS (1.33 ± 0.04) animals following a 1 month exposure to LPS compared to the other groups (1.76 ± 0.05, 1.74 ± 0.04, and 1.73 ± 0.05 for LPS, Adj, and control, respectively; *F* = 71.38, *p* < 0.001; **Figure 1C**). Nevertheless, it is interesting to note that animals that underwent LPS treatment for a month did not develop any significant deficit in LTP compared to control (**Figure 1C**). This result is indeed puzzling and it requires additional experiments to identify the possible causes of this effect. Remarkably, halting LPS treatment after a month did not restore LTP back to normal levels. Adj + LPS animals demonstrated deficits in expressing a full LTP following tetanic stimulation even at 1 week (**Figure 1D**), 1 month (**Figure 1E**), and 2 months (**Figure 1F**) after the LPS treatment has been stopped. In order to quantify the effects of duration of LPS treatment (factor a) and the different groups of animals exposed to them (factor b) we run a two-ways ANOVA (**Figure 1G**) which revealed an overall significant statistical difference for factor a (*F* = 23.24; *p* < 0.01) and for factor b (*F* = 65.22; *p* < 0.01) as well as a significant interaction between the two (*F* = 15.95; *p* < 0.01). In summary, these experiments show that a short exposure to LPS produces transient deficit in LTP expression in animals that were previously treated with Adj and in those that have received LPS only. LTP is restored back to normal levels if LPS exposure lasts for a week. A prolonged treatment with LPS in animals previously exposed to Adj induces persistent reduction in LTP that could not be re-established to normal levels even 2 months after the termination of the LPS treatment.

### CORTICOSTERONE REDUCES LTP IN SLICES OF ANIMALS UNDERGOING PROLONGED SYSTEMIC INFLAMMATION

**FIGURE 2:**
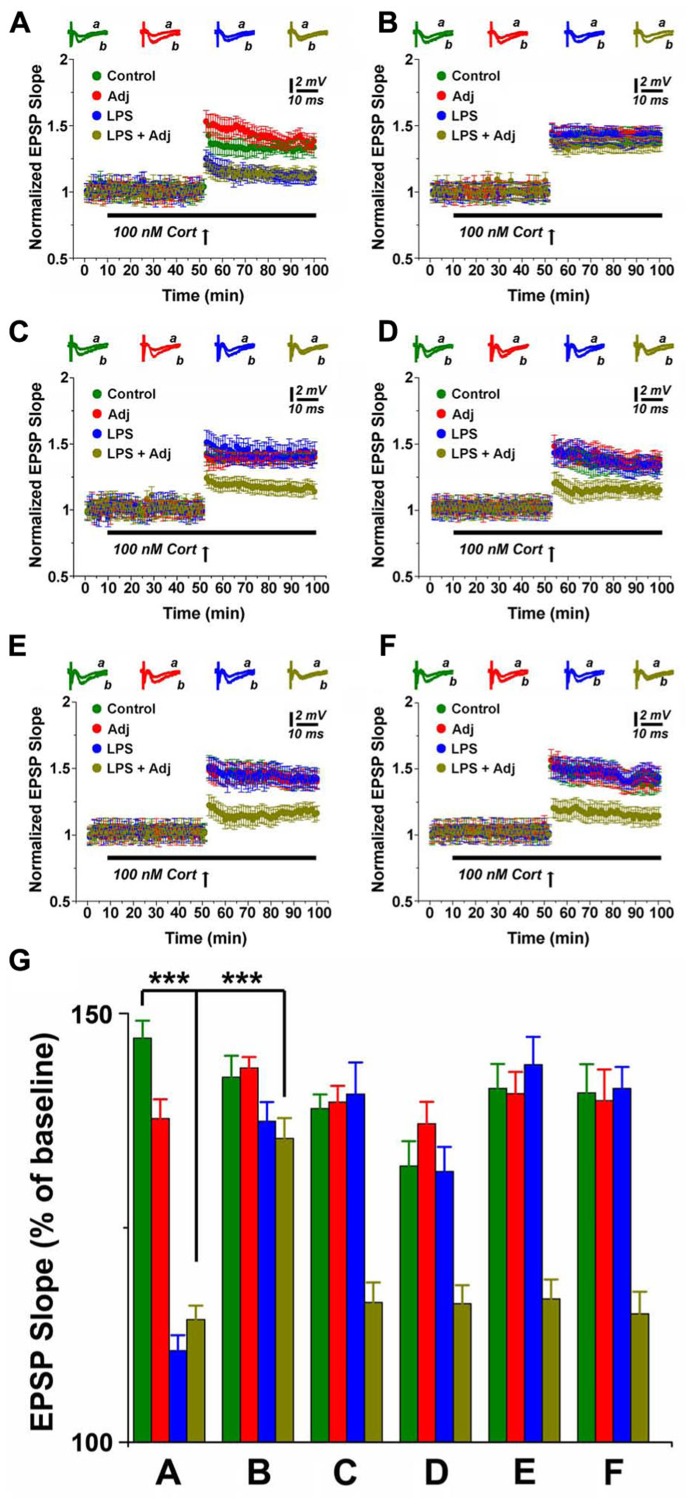
**CORT depresses LTP in hippocampal slices of animals undergoing LPS treatment of different duration.** LTP is highly impaired upon CORT exposures in slices from animals treated with LPS and LPS + Adj for a week **(A)**. Upon interruption of the treatment for a week LTP is restored at the control levels **(B)**. A persistent decrease in LTP is observed in slices from animals treated with LPS + Adj for a month **(C)** even after interruption of treatment **(D)** 1 week upon treatment interruption, **(E)** 1 month following treatment interruption, **(F)** 2 months following treatment interruption. **(G)** A combined analysis of all the data presented, conducted at 85 min after the tetanic stimulation. Further details of the results and the statistical comparisons are described in the Section “Results.” Statistical analyses were made with two way analysis of variance, followed by *post hoc* Tukey’s comparisons. In each panel of data, the arrow indicates the time of HFS delivery, the top traces are sample illustrations of original records before (a) and after (b) tetanic stimulation. ***p < 0.001.

Steroids have been considered a therapeutic option for patients undergoing severe, protracted inflammation (Patel and Balk, 2012; Sherwin et al., 2012). Moreover, even though activation of the stress response and release of steroids are known outcomes of prolonged inflammation (Wiersinga, 2011), no information is currently available on the effects of corticosterone (CORT) on synaptic plasticity in this setting. In order to tackle this issue, we investigated the consequences of CORT application on LTP in hippocampal slices of animals undergoing LPS treatment of different duration. Application of 100 nM CORT reduced LTP in control untreated animals (1.34 ± 0.04 compared to 1.77 ± 0.04 in slices from control animals not exposed to CORT, *p* < 0.001, **Figure 5**), an effect already reported by us (Maggio and Segal, 2007a) and other groups (Joels et al., 2008; Wiegert et al., 2008). Interestingly, however, application of CORT to slices of animals exposed to a short treatment with LPS resulted in even weaker LTP in LPS and Adj + LPS mice (1.10 ± 0.04 and 1.14 ± 0.04, respectively) compared to Adj and control groups (1.38 ± 0.05 and 1.34 ± 0.04, respectively; *F* = 74.21, *p* < 0.01; **Figure 2A**). Likewise, CORT exposure resulted in a LTP of lower magnitude compared to the one evoked in slices from LPS and Adj + LPS animals not exposed to CORT (**Figure 5**). These effects of CORT completely recovered 1 week upon interruption of LPS injections (**Figure 2B**). In contrast, however, CORT exposure of slices from animals treated with LPS for 1 month resulted in a persistent reduction of LTP. LTP was impaired in Adj + LPS animals (1.16 ± 0.05) following a 1 month exposure to LPS compared to the other groups (1.40 ± 0.08, 1.39 ± 0.03, and 1.38 ± 0.04 for LPS, Adj, and control respectively; *F* = 16.08, *p* < 0.001; **Figure 2C**). Remarkably, CORT still reduced LTP at 1 week (**Figure 2D**), 1 month (**Figure 2E**), and 2 months (**Figure 2F**) after the LPS treatment has been halted. In order to quantify the effects of CORT in groups of animals (factor b) which underwent to LPS treatments of different duration (factor a), we run a two-ways ANOVA (**Figure 2G**) test which revealed an overall significant statistical difference for factor a (*F* = 12.26; *p* < 0.01) and factor b (*F* = 92.86; *p* < 0.001) as well as a significant interaction between the two (*F* = 8.66; *p* < 0.01). In summary, these experiments show that (1) CORT further decreases LTP in animals undergoing a short or long exposure to LPS; (2) CORT effects on LTP are long lasting following a long LPS treatment; (3) CORT potentiates the effects of the systemic Adj + LPS injections on LTP reduction.

### MR ACTIVATION ENHANCES LTP IN SLICES OF ANIMALS UNDERGOING PROLONGED SYSTEMIC INFLAMMATION

CORT is known to modulate LTP through two different types of receptors: the MR and the GR. In order to study the contribution of each receptor to the effects of CORT on LTP, a specific combination of MR/GR antagonists in presence of CORT was applied to the slices from different groups of animals undergoing short and long LPS treatment. Bath application of RU38486 (RU, 500 nM), a blocker of GR, and CORT (100 nM) has been shown to reliably activate MR (Maggio and Segal, 2007a).

**FIGURE 3:**
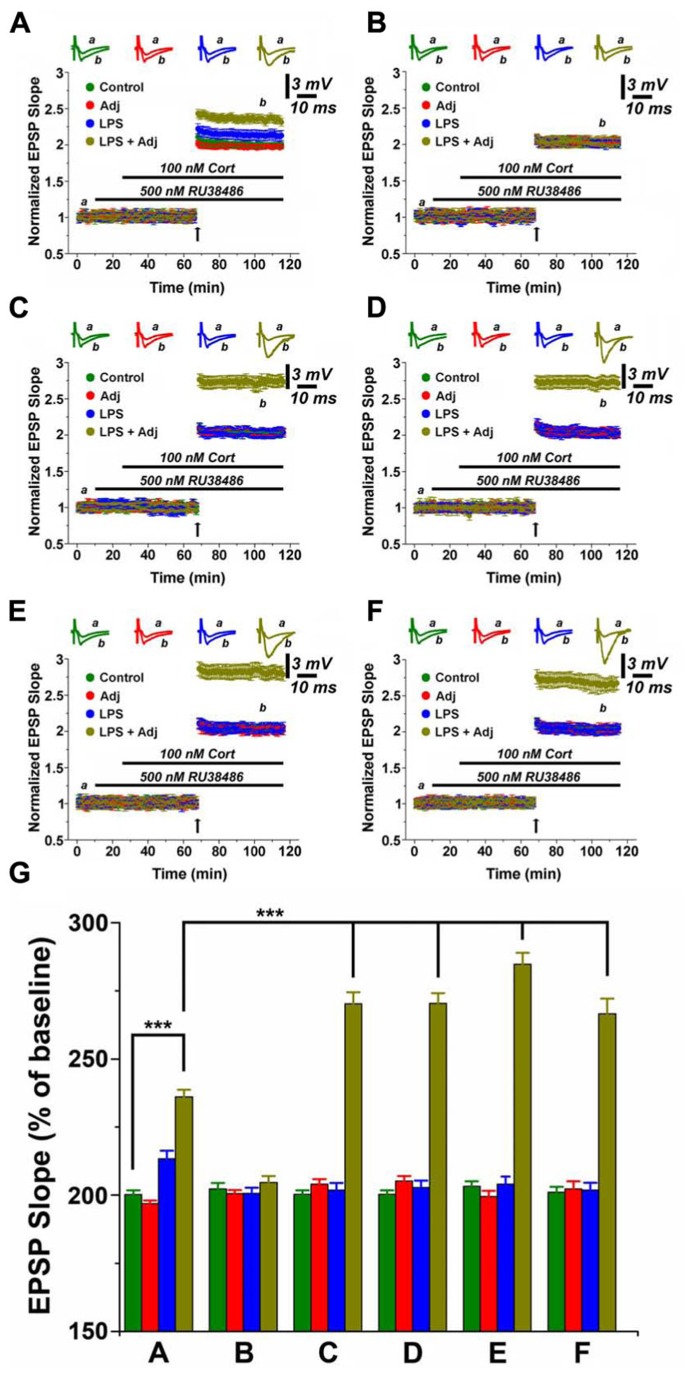
**MR activation enhances LTP in hippocampal slices of animals undergoing LPS treatment of different duration.** LTP is highly enhanced upon MR activation in slices from animals treated with LPS and LPS + Adj for a week **(A)**. Upon interruption of the treatment for a week LTP is restored at the control levels **(B)**. A persistent increase in LTP is observed in slices from animals treated with LPS + Adj for a month **(C)** even after interruption of treatment **(D)** 1 week upon treatment interruption, **(E)** 1 month following treatment interruption, **(F)** 2 months following treatment interruption. **(G)** A combined analysis of all the data presented, conducted at 100 min of recording. Further details of the results and the statistical comparisons are described in the Section “Results.” Statistical analyses were made with two way analysis of variance, followed by *post hoc* Tukey’s comparisons. In each panel of data, the arrow indicates the time of HFS delivery, the top traces are sample illustrations of original records before (a) and after (b) tetanic stimulation. ***p < 0.001.

Indeed, MR activation strikingly potentiated LTP in slices from Adj + LPS animals compared to the other groups. Following 1 week of LPS treatment, MR activation resulted in a short lasting LTP enhancement both in untreated animals as well as in Adj + LPS mice, notably with a stronger effect in the latter group (*p* < 0.0001; **Figures 3A** and **5**). Surprisingly, an LTP of greater magnitude was induced in slices of 1 month Adj + LPS mice upon MR stimulation. The delivery of a tetanus following application of RU and CORT resulted in a potentiation of 2.69 ± 0.09 in spite of 2.011 ± 0.05, 2.04 ± 0.04, and 2 ± 0.03 of LPS, Adj, and control, respectively (*F* = 153.91, *p* < 0.001; **Figure 3C**). This effect persisted at 1 week (**Figure 3D**), 1 month (**Figure 3E**), and 2 months (**Figure 3F**) upon halting LPS treatment. In order to quantify the effects of MR activation in slices of animals (factor b) undergoing to LPS treatments of different duration (factor a), we run a two-way ANOVA (**Figure 3G**). This test revealed an overall significant statistical difference for factor a (*F* = 31.62; *p* < 0.001) and for factor b (*F* = 586.12; *p* < 0.001) as well as a significant interaction between the two (*F* = 31.16; *p* < 0.001).

In brief, these experiments show that (1) MR activation enhances LTP in animals undergoing a short or long exposure to LPS; (2) MR effects on LTP are long lasting following a protracted LPS treatment; (3) MR activation reverses the reduction in LTP induced by systemic Adj + LPS injections.

### GR ACTIVATION INDUCES DEPRESSION OF SYNAPTIC TRANSMISSION IN SLICES FROM ANIMALS FOLLOWING PROLONGED SYSTEMIC INFLAMMATION

**FIGURE 4:**
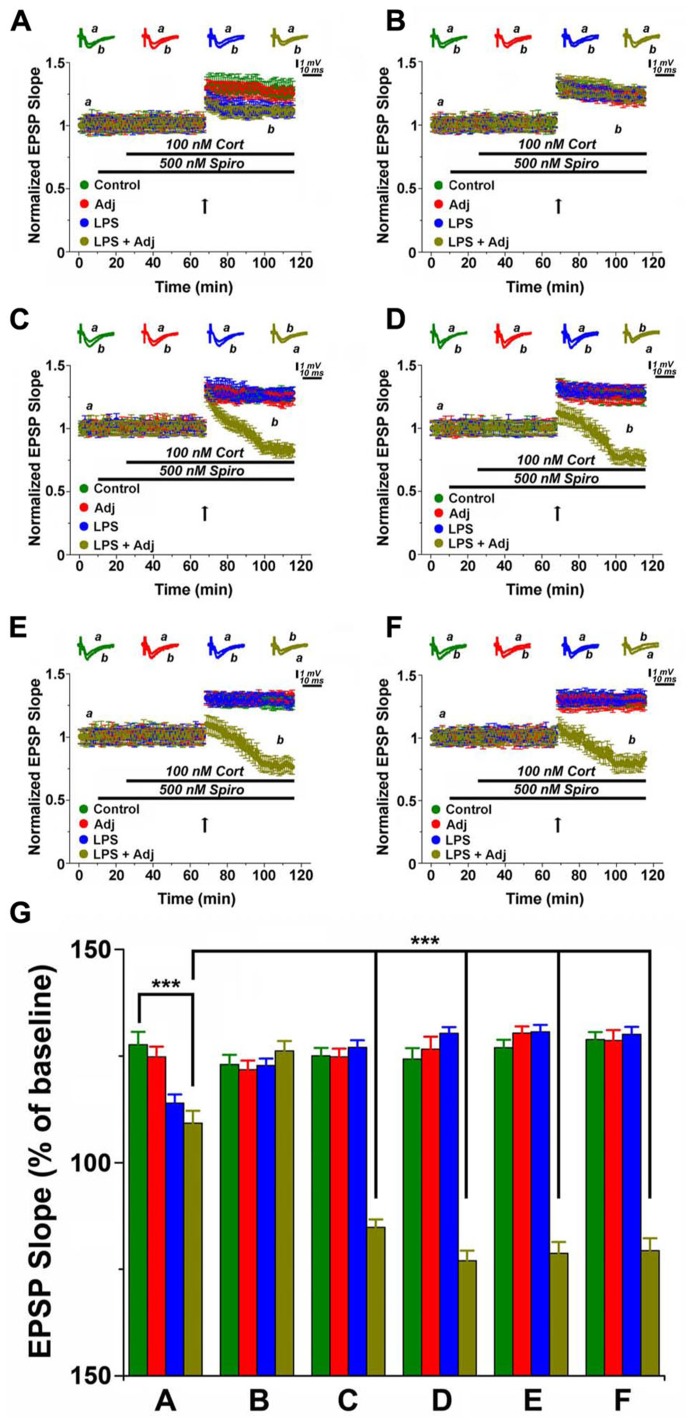
**GR activation converts LTP in slow onset depotentiation in hippocampal slices of Adj + LPS animals treated for a longer time period.** LTP is highly impaired upon GR activation in slices from animals treated with LPS and LPS + Adj for a week **(A)**. Upon interruption of the treatment for a week LTP is restored at the control levels **(B)**. A conversion from LTP to slow onset depotentiation is observed in slices from animals treated with LPS + Adj for a month **(C)** even after interruption of treatment **(D)** 1 week upon treatment interruption, **(E)** 1 month following treatment interruption, **(F)** 2 months following treatment interruption. **(G)** A combined analysis of all the data presented, conducted at 100 min of recording. Further details of the results and the statistical comparisons are described in the Section “Results.” Statistical analyses were made with two way analysis of variance, followed by *post hoc* Tukey’s comparisons. In each panel of data, the arrow indicates the time of HFS delivery, the top traces are sample illustrations of original records before (a) and after (b) tetanic stimulation. ***p < 0.001.

**FIGURE 5:**
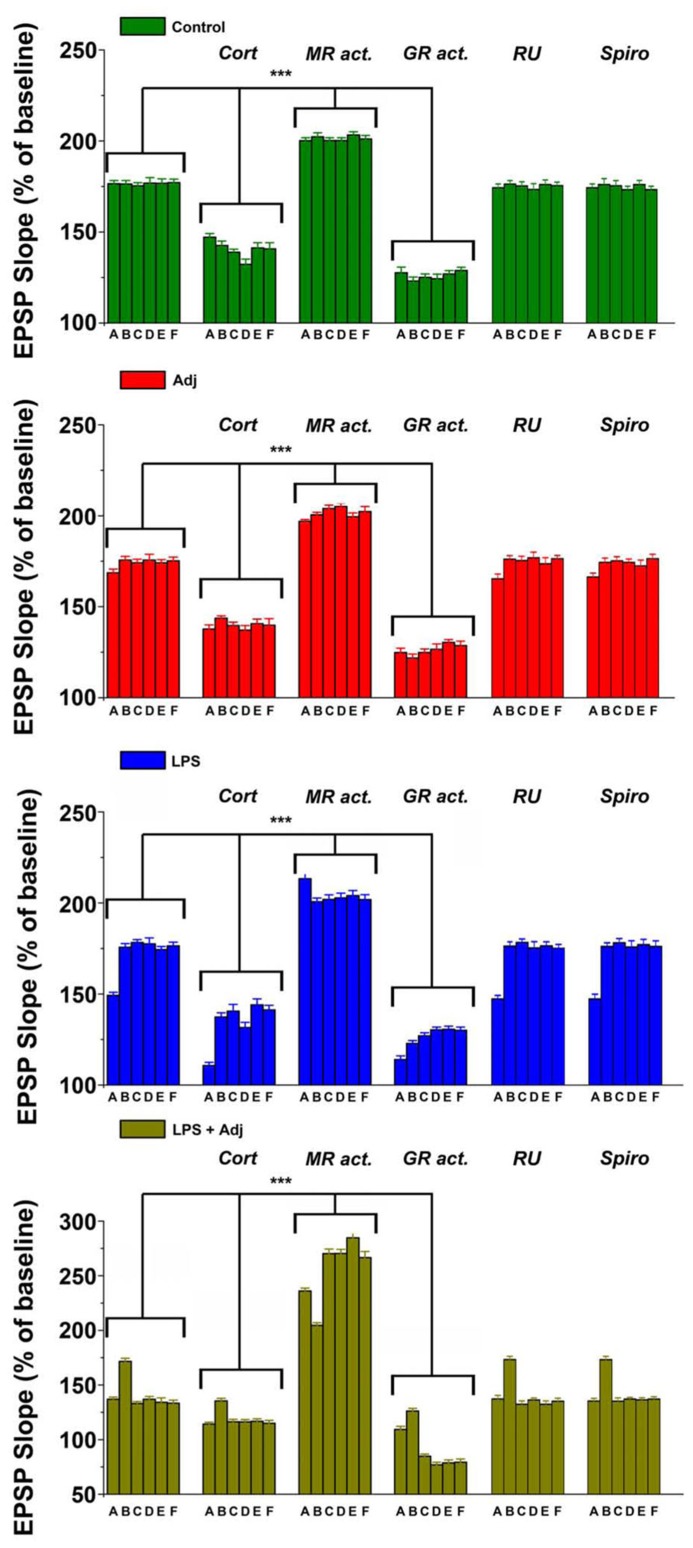
**Different animals groups express magnitude of LTPs upon different steroid receptor stimulation at different time points.** A combined analysis of all the data presented. Further details of the results and the statistical comparisons are described in the Section “Results.” Statistical analyses were made with two way analysis of variance, followed by *post hoc* Tukey’s comparisons. ***p < 0.001.

Bath application of Spironolactone (Spiro, 500 nM), an MR blocker, together with CORT (100 nM) activates GR (Avital et al., 2006; Maggio and Segal, 2007a). Stimulation of GR activation reduced LTP in slices of untreated animals, however a greater effect occurred in Adj + LPS treated mice (**Figures 4A** and **5**). Unexpectedly, GR were able to convert LTP in a slow onset depotentiation in slices of 1 month Adj + LPS treated mice: a tetanic stimulation delivered after washing in Spiro and CORT resulted in a reduced level of transmission in these animals (0.84 ± 0.04) compared to the other groups (1.26 ± 0.03, 1.25 ± 0.04, and 1.25 ± 0.04 of LPS, Adj, and control, respectively; *F* = 124.42, *p* < 0.001; **Figure 4C**). A similar effect was reproduced at 1 week (**Figure 4D**), 1 month (**Figure 4E**), and 2 months (**Figure 4F**) after discontinuation of LPS injections. A two-ways ANOVA quantifying the effects of GR activation in groups of animals (factor b) undergoing to LPS treatment of different duration (factor a; **Figure 4G**) detected an overall significant statistical difference for factor a (*F* = 7.98; *p* < 0.0001) and for factor b (*F* = 348.05; *p* < 0.001) as well as a significant interaction between the two (*F* = 29.97; *p* < 0.001). Summarizing, these experiments show that (1) GR activation reduces LTP in animals following a short exposure to LPS and (2) converts LTP in a slow onset depotentiation in animals treated for a longer period.

## DISCUSSION

In this study, we report the effects of peripheral inflammation on LTP and its modulation by steroids hormones. A major finding of our work is the evidence that systemic inflammation leads to different outcomes on LTP depending on its duration. Specifically, short lasting inflammation transiently weakens LTP while, in contrast, the exposure to LPS for a month disrupts LTP for a longer period in slices of animals that have previously received Adj. This effect persists even upon discontinuation of the LPS treatment. Although we have not addressed the possible mechanisms in charge of this phenomenon, based on current literature (Dilger and Johnson, 2008; Norden and Godbout, 2013) a possible explanation may consider the dual role of Adj both in “priming” the immune system (Staykova et al., 2008) as well as in promoting long lasting BBB breakdown (Rabchevsky et al., 1999). In this setting, following Adj treatment, a prolonged peripheral inflammation may release excessive concentrations of inflammatory cytokines, such as IL-1, IL-6, TNF-α, PGE2 in the CNS (Lynch et al., 2004; Dilger and Johnson, 2008) which may ultimately cause LTP impairment in these animals. In this respect, it is important to consider the long time frame of the systemic inflammation which indicates this phenomenon to be a chronic condition rather than an acute reaction to a noxious stimulus. It is therefore tempting to think that somehow a long lasting systemic inflammation may persistently modify brain functioning even after its remittance. Currently, however, there are no studies that have focused on such hypothesis. Needless to say, understanding the biological causes of this phenomenon seems of paramount importance to improve long term care of patients experiencing those conditions in order to prevent early onset of dementia (Barichello et al., 2005; Comim et al., 2009).

Another major finding of our study is the modulation of LTP by steroid hormones in animals undergoing LPS treatment. The effects of steroids seem to depend on the duration of the peripheral inflammation, with longer lasting effects in slices of animals undergoing a prolonged LPS treatment. This evidence shows that even if enduring inflammation has triggered persistent changes in the brain, a modification of such mechanisms is still possible, hence opening a window of opportunity for future intervention. Furthermore, the demonstration that steroids application in the slice is able to alter the outcomes of persistent inflammation shows that LPS-induced changes occur in the brain itself and are not an epiphenomenon of a condition taking place in the rest of the body.

Previous studies have suggested that CORT influences LTP in diverse manners according to the type of receptor (GR or MR) that has been activated (Joels and Krugers, 2007; Maggio and Segal, 2012b). In our experiments CORT reduced LTP in slices of Adj + LPS animals as well as in control untreated animals. This result is possibly due to a summation of the MR and GR activation in the slices, likely with a predominant GR effect. In fact, this present data as well as our previously published observations (Maggio and Segal, 2007a, 2009, 2012a,c) have shown that single activation of MR and GR, respectively either facilitated LTP or decreased it leading to a depotentiation of the network. Normally, the application of CORT in the slice results in the simultaneous stimulation of both receptors, however as intracellular MR are almost saturated at the baseline conditions (Maggio and Segal, 2012b), CORT likely causes an unbalanced response towards GR. Despite the mechanisms involved, it is interesting to look at our data in a clinical perspective. Steroids treatment has been shown to be beneficial in treating persistent inflammation (Batzofin et al., 2011; Sherwin et al., 2012), however, taken our findings in consideration, what would be their effect on cognition? Would they impair either the possibility to form new memories or recalling old ones in these patients? Furthermore, in patients recovering from prolonged sepsis, which role do steroids have in respect to the patient’s tendency in developing early cognitive impairment? These are interesting questions which have not been currently addressed. Indeed, additional behavioral experiments need to be performed in order to clarify these issues.

Activation of MR has been previously shown to enhance LTP in the hippocampus (Avital et al., 2006; Maggio and Segal, 2012a). In the current study, we successfully replicated previous findings, however in the context of prolonged systemic inflammation the effects of MR activation were even more striking than the ones previously reported. MRs are known to enhance LTP in the hippocampus through the activation of Voltage Gated Ca^2^^+^ Channels (VGCCs; Maggio and Segal, 2007a, 2012a). VGCCs have a higher threshold of activation therefore while on the one hand they may have a secondary function in inducing tetanic stimulation-evoked LTP, they may play a pivotal role in synaptic plasticity in situations where, upon challenging a network, a novel threshold for LTP is being set. MR activation may promote this change by affecting the inhibitory tone of the hippocampal network (Maggio and Segal, 2012a,c). Indeed more puzzling is the further enhancement in LTP following MR stimulation in slices of animals undergoing persistent inflammation. Two possible hypotheses may be put forward in order to explain this finding. In one case, the continuous inflammatory challenge may alter the inhibitory tone on pyramidal cells and enhance excitability of the slice. Alternatively, persistent inflammation may promote a homeostatic change of the network which through calcium stores (Maggio and Segal, 2007a; Vlachos et al., 2008, 2009; Segal et al., 2010) might be in charge of the MR dependent-additional enhancement of LTP. While the former theory does not seem to be likely (baseline responses under MR activation were not affected), the latter one looks more promising and additional experiments need to be conducted in order to better understand the molecular players in charge of this phenomenon.

It is interesting to consider what might be the role of MR in learning and memory and whether they would be able to reverse the cognitive changes induced by the persistent inflammation. Definitely, additional experiments should evaluate whether MR activation in the brain might have a preventive outcome in halting cognitive impairments following systemic inflammation.

In summary, our study shows that prolonged inflammation disrupts LTP in the hippocampus. A potential strategy aiming at MR activation in this setting might reverse the effects of inflammation on cognition as well as circumvents the deleterious effects of steroids on LTP.

## Conflict of Interest Statement

The authors declare that the research was conducted in the absence of any commercial or financial relationships that could be construed as a potential conflict of interest.
